# The future of sustainability in the context of COVID-19

**DOI:** 10.1007/s13280-020-01430-9

**Published:** 2020-12-07

**Authors:** Donna-Mareè Cawthorn, Alexandra Kennaugh, Sam M. Ferreira

**Affiliations:** 1 School of Biology and Environmental Sciences, University of Mpumalanga, Nelspruit, 1200 South Africa; 2 Oak Foundation, 43 Palace Street, London, SW1E 5HL UK; 3 Scientific Services, SANParks, Skukuza, 1350 South Africa

**Keywords:** Adaptive management, COVID-19, Emerging disease, Nationalist isolation, Totalitarian surveillance, Wildlife trade, Zoonosis

## Abstract

The COVID-19 pandemic is a global crisis emanating both from a virus (SARS-CoV-2) and from the drastic actions to contain it. Here, we reflect on the immediate responses of most world powers amid the pandemic chaos: totalitarian surveillance and nationalist isolation. Drawing on published literature, we consider measures such as wildlife-use bans, lockdowns and travel restrictions, along with their reverberations for people, economies and the planet. Our synthesis highlights significant shortfalls of applying command-and-control tactics in emergencies. For one, heavy-handed bans risk enormous unintended consequences and tend to fail if they lack legitimacy or clash with people’s values. Furthermore, reactive and myopic strategies typically view the pandemic as a stand-alone crisis, rather than unravelling the complex interplay of nature-society interactions through which zoonotic diseases originate. A return to adaptive management approaches that recognise root causes and foster socio-ecological resilience will be essential to improve human and planetary health and mitigate future pandemics.

## Introduction

The ongoing COVID-19 pandemic, caused by SARS-CoV-2 (Severe Acute Respiratory Syndrome Coronavirus 2), is likely the greatest crisis facing humanity since World War II (Kickbusch et al. 2020). Since identification of the first human infection in Wuhan, China, in late 2019 (Li et al. 2020), the disease has spread to over 200 countries, caused >39.5 million confirmed cases and claimed over 1.1 million lives worldwide (as of 18 October 2020; WHO 2020), with little sign of abating. Health care systems have consequently been stretched to their limits, resulting in rationing of scarce medical resources and precarious trade-offs on human lives (Emanuel et al. 2020). The devastating consequences of the pandemic, however, extend far beyond the immediate health crisis. Drastic government measures taken to ‘flatten the curve’ – including lockdowns, travel bans and militarised enforcement—have unravelled the fabrics of everyday life, while simultaneously crippling economies, impacting on human wellbeing, and impinging on people’s basic rights (Nay 2020; Nicola et al. 2020).

COVID-19 is the third coronavirus-related epidemic to emerge from a spillover from wild animals to humans, following SARS (Severe Acute Respiratory Syndrome) in 2003, and MERS (Middle East Respiratory Syndrome) in 2012 (Petrosillo et al. 2020). These concerns, coupled with COVID-19’s possible link with a Wuhan ‘wet market’ (Li et al. 2020), have reignited a worldwide debate about the potential human health threats posed by wildlife trade and consumption, and prompted several countries to take actions to halt the latter practices (Pinnock 2020; Standing Committee of the National People’s Congress 2020). While these threats are not in contention, the timing and justification of such actions are questionable, given early evidence that the virus is now spreading through human-to-human transmission (Li et al. 2020) rather than via repeated human-wildlife contacts.

Strategic government responses to the COVID-19 crisis dichotomise on two fronts: (i) totalitarian surveillance versus citizen empowerment, and (ii) nationalist isolation versus global solidarity (Harari 2020). Most world powers chose totalitarian surveillance and nationalist isolation, which essentially equate to command-and-control tactics. Centralised guidance from the World Health Organization (WHO) logically focuses on immediate actions to safeguard human lives and economies (Sohrabi et al. 2020). However, there is also a need for systemic thinking on feasible and sustainable long-term strategies for managing the pandemic, especially given the historically long incubation periods of similar coronavirus diseases, i.e., SARS, MERS. (Sahin et al. 2020). Overreliance of governments on high priority health guidance alone is likely to impose significant challenges to both socio-economic and ecological resilience—key elements of all-inclusive human wellbeing (Farley and Voinoy 2016).

Complex problems, like COVID-19, have inherent uncertainty and are best managed under ‘adaptive management’ frameworks that address the root causes of particular concerns (Gunderson et al. 2008). The current responses to COVID-19 address the symptoms (i.e. curbing emerging disease dynamics), while failing to confront the underlying causes (i.e. drivers of unsafe and unsustainable use of biological resources; global travel and economic connectivity that affect people and the environment at local scales). While command-and-control responses are appropriate for both simple tasks (e.g. measuring temperature) and complicated systems (e.g. constructing an aircraft), they are only useful in chaotic circumstances when dealing with large-scale perturbations in socio-ecological complex systems as a means of damage control (i.e. gaining control of symptoms). Returning to adaptive management approaches that acknowledge uncertainty and address underlying causes is an essential requirement for sustainable futures of complex socio-ecological systems. These approaches also recognise that the choices made today will have long-term repercussions for such systems. Against this backdrop, we speculate on what sustainable futures may realise in the COVID-19 aftermath. To gather evidence to reinforce our assessment, we used Boolean search operators to explore published and peer-reviewed literature indexed in the bibliographic databases Google Scholar, Scopus and Science Direct, and repeated similar searches in the ‘grey literature’ to retrieve relevant policy documents, working papers and other unpublished materials.

We begin our synthesis by reflecting on three primary responses to the underlying risks of relatively common zoonotic emerging diseases, a small fraction of which are associated with single-stranded RNA enveloped viruses like SARS-CoV-2 (Han et al. 2020). We further deliberate on the likely reverberations of choices already taken by many governments to contain the pandemic spread—totalitarian surveillance and nationalist isolation (Harari 2020). Our focus is on the direct and indirect repercussions of these choices for existing socio-economic frameworks, and within the wider milieu of other global environmental change drivers (e.g. climate change, habitat disruption, human migration etc.). We conclude by exploring new epistemological trajectories aimed at improving adaptive capacity and resilience, and thereby mitigating environmental mismanagement and emerging global health concerns.

## Responses to emerging diseases

### Minimise emerging disease opportunities

Totalitarian responses effectuated several immediate legislative changes relating to wild animals. These included widespread closures of ‘wet markets’ (Volpato et al. 2020) and blanket prohibitions on wildlife use (hunting, trading, consumption) in China (Standing Committee of the National People’s Congress 2020) and some African countries like Malawi and Gabon (Pinnock 2020), accompanied by voluminous calls to extend similar injunctions globally **(**Neupane 2020; Orenstein 2020). Such heavy-handed sanctions are unrealistic, inequitable and potentially self-defeating. This is firstly because the precipitous shut down of ‘wet markets’—which contribute substantially to urban and rural food security worldwide (Roe et al. 2020; Vandebroek et al. 2020)—was possibly premature, considering the hitherto unconfirmed links between the virus’s origin and the now infamous Huanan Market (Cohen 2020).

Secondly, history illustrates that such indiscriminate bans are seldom effective, since they fail to consider the centrality of wildlife in human livelihoods, the complexity of the trade, as well as the political, economic and social contexts in which they are implemented (Swan and Conrad 2014; Pooley et al. 2015; Bonwitt et al. 2018; Eskew and Carlson 2020). Contentious environmental management decisions that are likely to affect multiple actors and agencies require broad stakeholder engagement, transparent dialogue and reconciliation of diverse values and needs (Reed 2008). However, top-down rules that disregard this engagement, and conflict with the values and beliefs of those people expected to follow them, will almost certainly be met with resistance and distrust (Swan and Conrad 2014; Bonwitt et al. 2018). Indeed, there is little evidence that either zoonotic outbreaks or bans markedly deter wildlife consumption or trade (Mufunda et al. 2016; Seytre 2016; Bonwitt et al. 2018), including that of suspected disease hosts (Yang et al. 2007; Cronin et al. 2015; Akem and Pemunta 2020), and demand could even increase due to perceptions of scarcity (Conrad 2012). Where demand persists, there is considerable risk of driving activities deeper underground and enmeshing these with other organised criminal networks (Bonwitt et al. 2018; Eskew and Carlson 2020; Roe et al. 2020). It is precisely such circumstances that will hinder monitoring and regulation of the trade, promote unsanitary practices, and ultimately increase the potential for zoonotic outbreaks (Bonwitt et al. 2018; Roe et al. 2020).

Last, but by no means least, this myopic focus on wildlife trade as the single causative agent in emerging zoonoses overlooks the many other important anthropogenic and environmental drivers that amplify zoonotic risks. In fact, almost half of all human infectious disease outbreaks in recent decades have arisen due to changes in land use, agricultural activities, or other food production practices (Loh et al. 2015). Rampant deforestation, unbridled land conversion, intensification of farming, and infrastructure development have all expanded and modified the interface between wildlife, livestock and people, and created a ‘perfect storm’ for the spillover of animal pathogens to humans (Plowright et al. 2017; Faust et al. 2018). Once a spillover occurs, our hyper-connected global societies and transport systems make it easy for diseases to spread rapidly and transition into pandemics, as COVID-19 has tragically shown us. Crucially, if the world is to prevent similar devastating pandemics in the future, we urgently need to address all root causes of increasing zoonotic disease emergence, rather than focusing solely on wildlife trade.

### Minimise disease transmission mechanisms

Nationalist isolation aims to address the drivers of successful disease transmission by severely curtailing human movement. Along with domestic lockdowns, 100% of global destinations have implemented COVID-19 related travel restrictions, with most completely shutting borders, suspending flights and halting entry of non-citizens (UNWTO 2020). The architecture of global and societal connectedness is a key element of various economies (Bair 2008), the disruption of which carries major financial consequences. Trade in areas such as commodities, transport, distribution, and tourism (see Section 3) have been heavily affected by health-related restrictions and capital outflows (WTO 2020), and these shortfalls are unlikely to be recouped post-pandemic. COVID-19 has also placed extraordinary stress on food systems due to both supply and demand shocks, although some supply chains have demonstrated remarkable resilience to these stresses. In developing countries, where social safety nets are less well developed, the greatest food security threat has not been with the unavailability of food, but rather with the lack of access to food due to lockdown restrictions and financial constraints (OECD 2020). McKibbin and Fernando (2020) modelled the global macro-economic impacts of COVID-19 using various scenarios of differing infection intensities, but models reflecting consequences of different response options are rare.

Several commentaries suggest some potentially positive environmental outcomes owing to COVID-19 related restrictions, such as reduced air pollution and carbon emissions due to decreased domestic and international traffic (Neupane 2020). However, these improvements are transient and prone to reversal as restrictions are increasingly lifted. There will likely to be significant impacts on sustainability, and perhaps most notably on the environmental pillar of sustainability, if the global purpose is to restore socio-ecological systems to the way they were before the pandemic.

### Maximise immunity

Nationalist isolation endeavours to slow emerging disease growth (‘flatten the curve’) to enable medical facilities to cope with severe COVID-19 symptomatic cases, while also buying time for vaccine development (Harari 2020). This approach also prioritises medical capacity on COVID-19 responses, which inevitably compromises a range of other potentially fatal human health conditions (Emanuel et al. 2020). Nationalist isolation could theoretically suppress the dynamics of susceptible host populations to develop ‘herd immunity’ to SARS-CoV-2 (Randolph and Barreiro 2020), although the extent to which natural immunity persists after infection is yet to be confirmed, and there have been cases of reinfection (Fontanet and Cauchemez 2020; Long et al. 2020). While an effective vaccine represents the safest way to achieve herd immunity, lengthy clinical trials and cautious validation studies will hinder swift vaccine availability and thus broadscale vaccine control of COVID-19 (Kaur and Gupta 2020). An immediate and more probable scenario is additional waves of infections, most likely seasonal (Nickbakhsh et al. 2020; Smit et al. 2020). Even so, totalitarian surveillance and nationalist isolation are likely to remain as government preferred optimisation responses that allow adaption of the intensity of lockdown controls to oscillating levels of infection (Rawson et al. 2020).

The economic downturn and ‘exclusionary’ mentality associated with nationalist isolation further holds significant ripple effects on social structures and human wellbeing, including malnutrition, unpredicted criminal activity, breakdowns in informal family support systems, domestic violence, stigmatisation and xenophobia (Cheng 2020; Nicola et al. 2020). Totalitarian measures under this domain also extend to the imposition of lengthy and controversial alcohol and tobacco/nicotine bans in countries like South Africa, premised on the need to protect public health and strengthen individual immunity (Egbe and Ngobese 2020). However, much like wildlife-use bans, such prohibitions overlook the power of societal norms, as well as the grasp of addictions, and thus appear to be largely ineffective in breaking people’s long-term habits. Although well intended, they served to undercut legal excise tax revenues and fuelled a burgeoning black-market trade for these products (van Walbeek et al. 2020) that might well prove more difficult to extinguish than the virus itself.

### Suppositions

The present command-and-control reactions are responding to the constraints of the current operating framework. Returning to adaptive management once the pandemic chaos has been brought under control may require decision-makers to reimagine new systems and to reflect on what could have been done differently. The pandemic is also forcing humankind to address the underlying assumptions in our present models for living. The way that nature is rebounding in a new quieter world, as noted through numerous anecdotal natural history observations accumulated globally through social media, is a wake-up call to our dysfunctional relationship with the planet and a reminder that humans are just one species in the system. Recognising that human health is intricately linked with planetary health will be critical if we are to emerge stronger after the crisis.

## Futuristic outlooks for global change patterns and existing frameworks

Several consequences of the pandemic will impose changes on the existing global socio-economic order. We elaborate on four aspects. The first is the totalitarian call to shut down ‘wet markets’ and to abolish the global wildlife trade in its entirety (Neupane 2020; Orenstein 2020; Pinnock 2020; Standing Committee of the National People’s Congress 2020), which risks exacerbating inequality and poverty with no commensurate benefits. The totalitarian surveillance regime has also seen some authorities deploying hi-tech digital technologies to monitor their citizens (e.g. face recognition software, smartphone intelligence, biometric data collection), reputedly for the purpose of contact and disease spread tracing. Several commentators highlight the impacts of such intrusions on human privacy and free choice (Harari 2020; Ienca and Vayena 2020). Other measures taken—such as mandatory COVID-19 testing, involuntary quarantines and enforced hospitalisations (Parmet and Sinha 2020; Weiner 2020)—might be considered similarly extreme and intrusive on human rights.

The second aspect relates to the global ‘paralysis’ caused by the sustained nationalist isolation restrictions on travel, which have possibly only had modest effects on the pandemic trajectory (Chinazzi et al. 2020). These constraints have, however, had unprecedented impacts on aviation and tourism industries that crucially depend on both international and national visitors (Gössling et al. 2020; Nicola et al. 2020), with losses of up to $5.5 trillion and 197 million jobs projected for the sector worldwide (WTTC 2020). Trial health screening initiatives at London’s Heathrow Airport—including facial recognition thermal screening technologies, UV sanitation and contactless security screening equipment—also provide a glimpse of how travel standards might change in the near future (Bates 2020). Even so, lingering pandemic fears and the associated global recession will likely keep global travel substantially reduced for some time.

The third is the impact on other industries and commerce. Many businesses, and particularly large corporations, have been able to overcome the challenges of human-movement restrictions by diverting to online communication technologies, while trade products continue to move internationally (Craven et al. 2020). Others have not been so fortunate (Parmet and Sinha 2020), and many have closed. A by-product of nationalist isolation will likely be the growth in remote stations of working.

Lastly, it is likely that the effects of nationalist isolation, along with the COVID-19 related disruptions in global food supply chains, will create an increased demand and dependence on locally produced products. This could help hard-hit economies to offset the impacts of the global recession, while also reducing vulnerability to future external food shocks or disruptions.

We discuss these consequences further in the context of prevailing conservation models and impinging global environmental change drivers.

### Conservation models

Two competing ideologies exist in contemporary wildlife conservation approaches: An exclusive, biocentric or animal protectionist ethic; and an inclusive, anthropocentric, or human rights-orientated one (Swan and Conrad 2014; Madzwamuse et al. 2020). Exclusive ideologies underpin the non-consumptive eco-tourism model that abounds across the globe (Gössling et al. 2020). Substantial reductions in travel predictably translate to substantial drops in eco-tourism as a key economic basis of conservation models. For example, the iconic African experience of the wildebeest migration in the Serengeti National Park typically attracts large numbers of foreign visitors each year and contributes significantly to Tanzania’s economy (Gardner 2016). The global curtailment of travel inevitably collapsed this economic stimulus, while also cutting funds for wildlife protection.

Even in the absence of nationalist isolation travel restrictions, many exclusive animal-rights conservation organisations rely on donor-based funding to accommodate non-consumptive ideals. Nevertheless, both public goodwill and the philanthropic super-rich are likely to prioritise support for controlling COVID-19 over that for wildlife protection in the foreseeable future (Neupane 2020), especially if exclusive ideologies continue to separate human social resilience from ecological resilience.

Inclusive human rights ideologies promote the sustainable use of all values of ecological resources, including consumptive values. African countries embrace this ideology the strongest (Madzwamuse et al. 2020). For instance, Botswana recently reinstated elephant hunting as part of the wildlife use spectrum, following inclusive consultation with its citizens (Cassidy and Salerno 2020). Inclusive ideologies are as much at risk of a global downturn in travel because a large fraction of the non-consumptive eco-tourism values cannot realise into economic return. African conservation strongholds, such as the Kruger National Park, are key revenue generators through wildlife viewing (Chidakel et al. 2020) and provide direct and indirect employment for thousands of people, including many from vulnerable rural communities. The closing of parks during nationwide lockdowns nevertheless severed these revenue streams and imperilled countless jobs and lives. Parks that embrace inclusive human rights ideologies and thus recognise sustainable use of all values may have broader options of establishing funding resilience through, for instance, complementing fragile tourism-based incomes with less fragile consumptive-use incomes (Lindsey et al. 2020).

Although inclusive conservation ideologies promote consumptive use, they will embed in a moral complex trade-off of food security versus zoonotic health risks. Throughout the developing world, ‘bushmeat’ (or wild meat) and other wildlife resources represent a crucial and irreplaceable source of food, income, medicine and cultural identity for hundreds of millions of vulnerable people (Cawthorn and Hoffman 2015; Nunes et al. 2019; Friant et al. 2020), the value of which is often amplified during periods of hardship (Brashares et al. 2004; de Merode et al. 2004). At the same time, zoonosis is relatively common and increasing (Han et al. 2020). Nevertheless, strictly enforcing totalitarian proscriptions on wildlife use, particularly amid unaddressed social inequalities and few alternative livelihoods, would have a disproportionate and devastating impact on the food security and wellbeing of myriads of people, potentially plunging them deeper into poverty and criminality (Bonwitt et al. 2018). Of course, the global demand for wildlife products is not only a response to food and livelihood insecurity, but also one tied to luxury, elitist consumption (Drury 2011; Volpato et al. 2020). These two issues undoubtedly need to be tackled differently and appropriately; however, imposing legal sanctions to induce behaviour change in an emergency context (Bonwitt et al. 2018) is unlikely to be the solution in either scenario.

An alternative to indiscriminate bans would be to enhance regulation of wildlife commodity chains, particularly those involving live animals (Roe et al. 2020). This might entail devising protocols for the hygienic handling, butchering and processing of wild animals; improving health, sanitation, traceability and surveillance systems along supply chains; as well as taking steps to reduce human contact with high-risk species (Bonwitt et al. 2018). These measures would likely require complementary awareness campaigns that inform and educate consumers on the risks of zoonotic transmission and the consequences of food choices and habits (citizen empowerment), thereby accommodating and incentivising human behaviour change towards cautious, regulated and safe wildlife use (Bonwitt et al. 2018; Volpato et al. 2020). Such initiatives might also then encourage people to use wild resources more sustainably and with respect. Responses within an inclusive human rights conservation ideology could help integrate social resilience with ecosystem resilience, a key element of sustainability.

### Global environmental change drivers

**Fig. 1:**
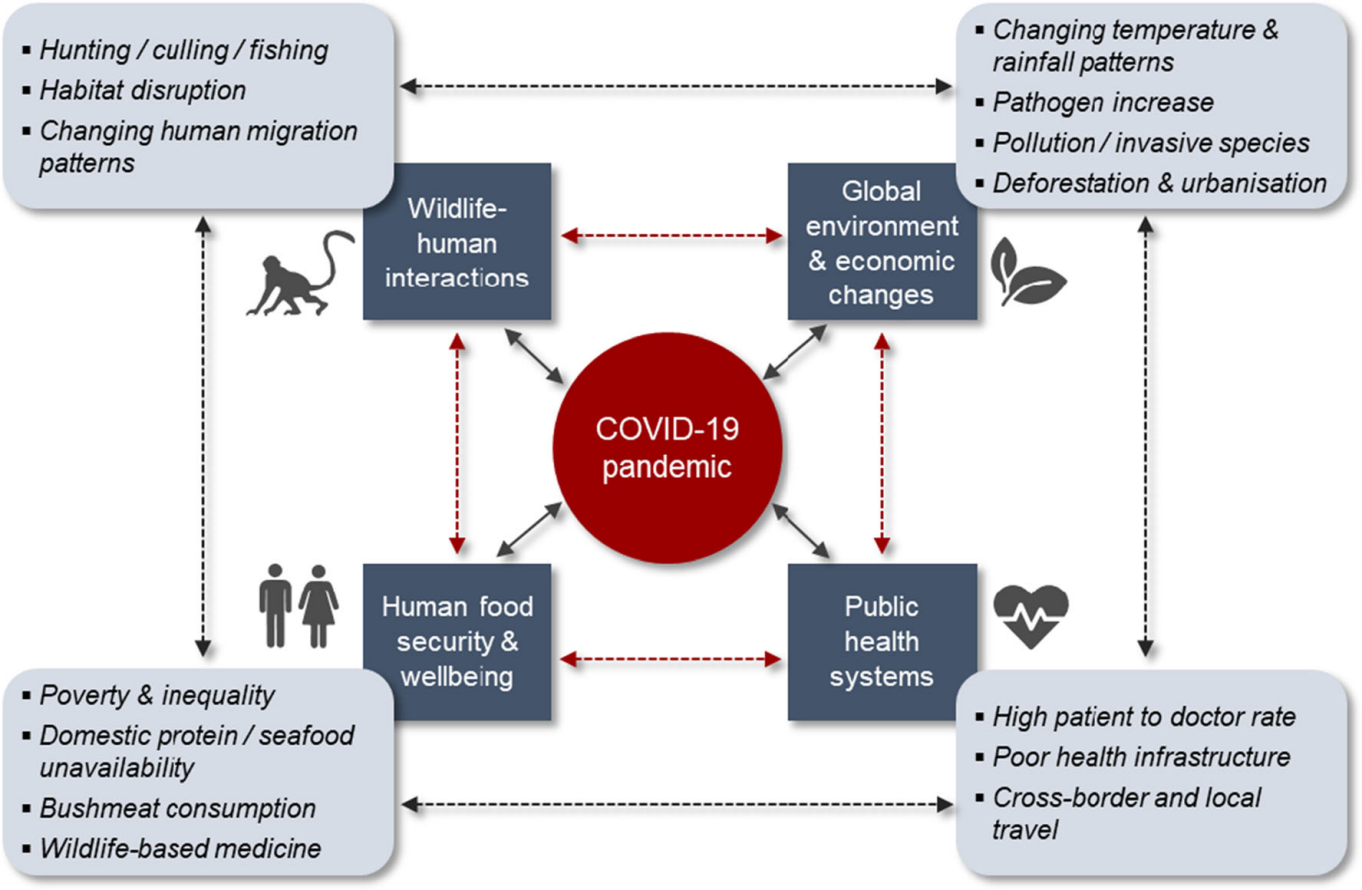
Conceptual links in human-nature interactions and the repercussions for COVID-19 and global public health. Modified from Khan and Sesay (2015)

The current pandemic is a symptom of a complex interplay of political, economic, social and environmental factors that collectively compromise planetary health and thus also human health (Fig. 1). Emerging disease is just one of numerous global environmental change drivers that affects socio-ecological resilience (Sala et al. 2000). The harvesting and trade of wild animals, whether legal or illegal, undoubtedly contributes to this risk, and may also jeopardise the persistence of many species that humans benefit from. Along with wildlife commodification, human encroachment on wildlife areas (e.g. through agricultural expansion, logging, mining and urbanisation) and resultant habitat disruption are also important risk factors for zoonotic spillover, as well as biodiversity loss (Johnson et al. 2020; Roe et al. 2020). Several other inter-related environmental change drivers can also have knock-on effects. For instance, climate change potentially influences the lifecycle of pathogens that affect human wellbeing, while also holding consequences for terrestrial and seafood production systems (Khan and Sesay 2015). Shortages of seafood or alternative domestic proteins, in turn, can accelerate bushmeat hunting and market sales (Brashares et al. 2004). Furthermore, pollution and invasive species degrade ecosystem services, such as clean water and fresh air. All these detectable direct effects originate from present global economic development frameworks, which also accentuate human inequalities and drive socially disruptive activities like crime (Braithwaite 2013). Moreover, most reactive strategies aimed at mitigating global change impacts on infectious diseases, as well as those taken to control zoonotic outbreaks, tend to consider single causal factors rather than elucidating on the complex nature-society interactions (Fig. 1). Unpacking these various factors and their inextricable links with human livelihoods, institutional structures and decision-making processes would foster an enhanced understanding of early warning signals, adaptive responses, and post-crisis management measures (Khan and Sesay 2015).

The global response to COVID-19 highlights some important lessons. Firstly, it is useful and desirable to respond to change drivers aggressively and fast. Secondly, it demonstrates that authorities are indeed capable of swiftly implementing policies and programmes if they believe that the risks of inaction outweigh the costs. For instance, several developing countries established basic infrastructure services like water and sanitation systems in a matter of weeks (Arakpogun et al. 2020), initiatives that had previously been procrastinated for decades of political wrangling and corruption. A third lesson associates with ill preparedness of current operating systems for future scenarios that are not so directly detectable. For example, it is not easy to address a climate change threshold that collapses pollinators, the key element to human food security, by nationalist isolation or totalitarian surveillance. Credible climate change projections (Gillard et al. 2016) already illustrate that humankind likely requires global solidarity and citizen empowerment. These shifts, however, will require re-design of the present global economic model – a seed of political and financial unease for world leaders.

## Opportunities and new frameworks

Our synthesis reflects on several COVID-19 responses and their possible ripple effects. All of the former, however, are reactive and entrenched within existing world order frameworks. The pandemic highlights that people lack a system that allows for dynamic calibration between use and abuse of biological diversity. The antiquated existing economic frameworks and governance systems struggle to accommodate this requirement. It is thus not surprising that the present global response is reactive.

System disruptions provide opportunities to implement a systems re-design, a principle at the heart of the cycle of adaptive approaches to managing complex socio-ecological systems (Gunderson et al. 2008). This means that it will not be sensible to go back to ‘business as usual’ with just a few minimal changes. New frameworks may require complete overhaul using techniques embedded in second-order logic (e.g. dynamic stochastic equilibrium modelling; Dilaver et al. 2018). The COVID-19 pandemic necessitates assertive adoption of complexity theory (Manson 2001), resting on principles of good inclusive governance (Lockwood et al. 2010). Embracing interdependency of social and ecological systems through complexity adoption should introduce resilience that brings about a beneficial reduction of fragility (Besedeš et al. 2016). Calls for conservation bailouts that enhance socio-ecological resilience highlight that governments can invest financial support to redesign the present fragile system exposed by the COVID-19 pandemic (McCleery et al. 2020). Mutualism is key, with ideas and leadership fostering sound risk management. That which remains fragile is likely to have an asymmetric response to volatility in various global environmental change drivers and other stressors (Sala et al. 2000). Generally, such volatility will do more harm than good, as exemplified by the present system’s response to the pandemic (Harari 2020).

A new world order will most likely rediscover dependency on locally produced products (Vandebroek et al. 2020), an outcome that could substantially boost developing country initiatives to restore and grow domestic economic structures. This will create opportunities to develop policies promoting self-sufficient and sustainable local natural resource economies as part of the reboot stimulus packages. A new world order, however, will need to draw on the lessons from the pandemic to develop risk responses and scenarios that mitigate the consequences of the nationalist isolation and totalitarian surveillance global leadership decisions taken during the outbreak (Harari 2020). It is notable that some governments have already entrenched environmental policies and circular economic plans into their pandemic recovery strategies (Camilleri 2020), an expression of a solidarity process.

Citizen empowerment and global solidarity (Harari 2020) will be key elements if the world is to transition to new approaches. Although the COVID-19 crisis highlights several risks associated with the present global responses, coherent analyses of economic, political and social influences on sustainability in the future could highlight the costs and benefits of totalitarian surveillance and nationalist isolation responses. Even so, escaping the perceived security of the old order on monetary and political dictations required to re-design aspects will prove most difficult. As devastating as the current pandemic is, it might just provide the impetus to stimulate this escape.
